# Haploidentical Stem Cell Transplantation in Adult Haematological Malignancies

**DOI:** 10.1155/2016/3905907

**Published:** 2016-05-30

**Authors:** Kevon Parmesar, Kavita Raj

**Affiliations:** ^1^Department of Haematology, Kings College Hospital NHS Foundation Trust, Denmark Hill, London SE5 9RS, UK; ^2^Kings College London, Kings College Hospital NHS Foundation Trust, Denmark Hill, London SE5 9RS, UK; ^3^Department of Haematology, Guys and St. Thomas' NHS Foundation Trust, Great Maze Pond, London SE1 9RT, UK

## Abstract

Haematopoietic stem cell transplantation is a well-established treatment option for both hematological malignancies and nonmalignant conditions such as aplastic anemia and haemoglobinopathies. For those patients lacking a suitable matched sibling or matched unrelated donor, haploidentical donors are an alternative expedient donor pool. Historically, haploidentical transplantation led to high rates of graft rejection and GVHD. Strategies to circumvent these issues include T cell depletion and management of complications thereof or T replete transplants with GVHD prophylaxis. This review is an overview of these strategies and contemporaneous outcomes for hematological malignancies in adult haploidentical stem cell transplant recipients.

## 1. Introduction

Over 50 years ago, it was first demonstrated that total body irradiation (TBI) along with transplantation of genetically identical (syngeneic) bone marrow could induce remission in a minority of patients with end-stage leukaemia [1]. Whilst transplantation was initially limited to bone marrow obtained from an identical twin, later identification of HLA types made the process of allogeneic transplantation possible that is from nonidentical HLA-matched donors such as siblings [2]. Subsequently, allogeneic transplantation was shown to be curative in a small percentage of patients with acute leukaemia who, at that time, were deemed incurable [3]. This was an especially significant outcome, despite frequent setbacks such as aggressive leukaemia progression and posttransplant complications like infection and graft-versus-host disease (GVHD) [4].

Further efforts were therefore focused on exploring how the procedure could become more successful in a greater number of patients. It was later established that transplants were more effective during the first remission of leukaemia, when transplantation could achieve a cure in more than 50 percent of patients [3, 5]. It was also found that patients who suffered subsequent GVHD had a better leukaemia-free survival in the long term [6]. This has now been determined to be part of a graft-versus-tumour effect (graft-versus-leukaemia or GVL effect) in which allogeneic immune cells eliminate occult tumour cells which may have survived the initial conditioning [7, 8].

Even more recently, advances in transplantation techniques have led to improved survival rates and reduced incidence of complications such as GVHD, thus lowering rates of transplant-related morbidity and mortality [9]. These include improved preparative regimens such as reduced intensity conditioning (RIC), which causes less severe side effects whilst still ensuring transplant engraftment [10]. RIC has also enabled transplantation in older, more comorbid populations, where myeloablative (MA) conditioning would have led to more substantive harm. Other techniques used involve better informed measures to prevent or limit GVHD and techniques to reduce the risk of posttransplantation opportunistic infections [4].

Transplantation has now been extended successfully to include HLA-matched unrelated donors with the development of national bone marrow registries in over 50 countries worldwide [4]. Studies have shown that, in some cases, fully matched unrelated donor (MUD) transplants can be comparable with matched related donors (MRD) in terms of disease-free survival and overall survival [11, 12]. Umbilical cord blood has also been identified as a source of haematopoietic stem cells (HSCs) for transplantation [7].

Haematopoietic stem cell transplantation (HSCT) is now a well-established treatment option for conditions such as acute myeloid leukaemia (AML) and myelodysplastic syndromes (MDS), as well as a number of other blood disorders [13]. In European centres alone, close to 15,000 allogeneic transplants were performed in 2013 and this number is increasing annually [14].

## 2. Limitations of HLA-Matched Transplants

Unfortunately, as few as 30 to 35 percent of patients will have an HLA-identical matched sibling donor available for HSC donation [7]. Furthermore, despite an estimated 25 million HLA-typed potential volunteer donors on the worldwide register [15], it remains difficult for some patients to find timely unrelated donors. This problem is most significant for persons of ethnic backgrounds that vary from the donor pool and persons of mixed heritage. It has been estimated that the chance of success in finding a matched donor ranges from 79% of patients with Caucasian background to less than 20% for some ethnic groups [16]. This is due to a variety of factors, including greater HLA polymorphism among persons of ethnic minorities, a smaller pool of potential donors, and higher rates of attrition from donor registries [17, 18].

Additional difficulties arise when a transplant is needed urgently, for example, in the case of particularly aggressive or rapidly progressing disease. The search for a transplant can often be a lengthy process involving identification, typing, and collection of cells from the stem cell donor. The entire process has been estimated to take a median of 4 months [9]. Shockingly, retrospective data have shown that even after a matched donor is found, only 53% of transplants actually proceed with delays and resultant disease progression being a major factor preventing follow-through [19].

Umbilical cord donations can solve many of these issues, mainly through reduced search times and greater mismatch tolerance [7]. Unfortunately, cord blood produces very few HSCs and therefore double cords may be necessary to provide adequate HSCs in adult patient [20]. The availability of cords from accredited banks is also a significant limiting factor. Engraftment time may be prolonged compared to regular HSCT, leading to prolonged neutropenia and subsequent susceptibility to infection in the posttransplant period. UCBT therefore results in higher rates of posttransplant complications and higher overall transplant-related mortality [9, 21].

## 3. Haploidentical Transplants as an Option

An alternative option is the transplantation of stem cells from a related donor who is only partially HLA-matched [22]. The genes on chromosome 6, which encode HLA antigens, are very closely linked, and, as a result, a child is likely to inherit one full set of genes from each parent. Each set is referred to as a haplotype. Whilst there is only a 25% chance of siblings sharing the same two parental haplotypes, it is significantly easier for patients to find a family member who is matched fully with only one of the HLA haplotypes (with the other being different). A transplant of this type would be referred to as a haploidentical transplant [23].

Haploidentical transplants offer substantial benefit to patients who have difficulty finding a matching donor, as nearly all patients will have an available haploidentical parent, sibling, child, or other relatives [9]. Haploidentical transplants can therefore improve access to transplantation, especially in ethnic variant patients who may find it near impossible to secure a matching donor. One report has estimated that over 95% of patients can find at least one haploidentical donor, with the average patient having 2 options or more [24].

Haploidentical transplants may provide more choice in donor selection in terms of age, cytomegalovirus status, and ABO compatibility [25]. It also allows easy access to posttransplant cellular therapies like donor lymphocyte infusions, if necessary [21]. Importantly, in case of graft failure, it provides the opportunity for a second graft from the initial donor, or from another family member who is available as an alternative donor.

The immediate availability provided by haploidentical transplants can provide further benefit by reducing associated costs and delays of finding unmatched donors (as described above), thus helping patients who may need a transplant urgently and creating opportunities for many more. These transplants can have a special role in less wealthy countries, where volunteer donor registries may not exist or where cost might be a prohibitive factor for MUD transplants, which are typically more expensive [26].

## 4. Haploidentical Transplant Strategies

The effects of transplantation with HLA mismatch had been established very early, when researchers sought to determine the acceptable limits to which a mismatched transplant could still be completed successfully. It was found that, compared to patients who had fully matched donors available, patients who underwent haploidentical transplants after myeloablative conditioning (in the form TBI) had higher rates of graft rejection, with the extent of HLA mismatch predicting the incidence risk of graft failure [27]. Another noted complication was acute severe GVHD which developed more often and sooner after transplantation [28]. Mismatched patients had a higher (70 percent) chance of developing GVHD of grades II to IV, which occurred at a median of 14 days, compared to a 42 percent incidence at a median of 22 days for the fully matched control group.

Whilst the intense bidirectional alloreactivity to incompatible HLA molecules was clearly a major limitation to these transplants, it had been hypothesised at that point that the manipulation of donor T-lymphocytes in the transplanted marrow could alleviate some of the effects of graft rejection and GVHD. Prior studies had shown that T cell depleted transplantation reduced rates of GVHD but increased rates of graft failure, potentially due to immunologic rejection via residual recipient T-cytotoxic lymphocyte precursors with antidonor specificity [29]. On the other hand, transfusion of “buffy coat” peripheral lymphocytes reduced graft rejection but led to a higher incidence of chronic GVHD, likely due to activation of effector T cells against host tissue [30].

In order to circumvent these problems, strategies centred around two major modalities [21]. Firstly, the transplant could be depleted of T cells using one of several techniques, followed by various measures taken to improve engraftment rates and reduce infectious complications. Otherwise, the transplant could be T cell replete, but with measures taken to reduce the risk of GVHD. A number of these strategies and their corresponding biological rationales are detailed below.

### 4.1. T Cell Depleted Transplantation

#### 4.1.1. T Cell Depletion with Megadose of Positively Selected CD34+ Progenitors

The earliest attempt focused on T cell depleted transplantation in patients with end-stage chemoresistant leukaemia and involved the use of an extremely myeloablative, immunosuppressive conditioning regimen (using 8 Gy unfractionated TBI, 50 mg/kg ×2 cyclophosphamide, rabbit anti-thymocyte globulin 25 mg/kg (ATG), and 10 mg/kg thiotepa) in order to assist engraftment [31, 32]. Key to overcoming the HLA mismatch was the infusion of a “megadose” of HSCs obtained by adding granulocyte colony stimulating factor (G-CSF) mobilised stem cells to bone marrow cells that were T cell depleted by soybean agglutinin and E-rosetting. This rationale was based on numerous preclinical studies that achieved high rates of engraftment using large doses of T cell depleted bone marrow. Preliminary follow-up showed a high engraftment rate (16 out of 17 patients engrafted successfully), and overall the regimen resulted in engraftment for 80 percent of patients with only 18% incidence of acute GVHD and no chronic GVHD reported.

Further modifications included the purification and positive immunoselection of CD34+ HSCs, in addition to further depletion of T cells and also B cells (to prevent EBV-related lymphoproliferative disorders) [33]. Fludarabine then replaced cyclophosphamide, the dose of TBI was reduced, and posttransplant G-CSF was removed from the protocol. Overall, 255 patients with acute leukaemia were treated, with engraftment rates of 95% and very low rates of both acute and chronic GVHD (only 5% of patients treated under the revised regimen suffered from acute GVHD grade II or higher).

These results confirmed that a megadose of CD34+ HSCs could overcome histocompatibility barriers by “veto activity,” where a group of cells has the ability to specifically inhibit a cytotoxic T-lymphocyte precursor (CTLp) cell response, against antigens presented by those veto cells [21, 34]. It is thought that CD34+ cells, or a group of cells comprising part of the CD34+ megadose, could veto any residual antidonor CTLp activity that had previously acted to reject the graft. These carefully designed alterations to the graft demonstrated clearly that, by depleting T cells to less than 2 × 10^4^/kg sufficiently, GVHD can be effectively prevented [33]. It is noteworthy that no posttransplant immunosuppression was used in these patients, giving the added benefit of lower rates of leukaemia relapse.

The Achilles heel of this carefully constructed plan was delayed immune reconstitution resulting in nonrelapse mortality (NRM) as high as 57%, particularly due to opportunistic infections by viral and fungal pathogens. In response to this threat, donor T cell clones were raised in vivo against CMV and* Aspergillus* antigens, screened to be non-cross-reactive to the recipient, and were effective as posttransplant immunotherapy in doses up to 1 × 10^6^/kg. Additional analyses of these patients highlighted that NK alloreactive donors impacted favourably upon the survival of patients with AML patients transplanted in CR with event-free survival of 67% compared to 18% in those without an NK alloreactive donor.

#### 4.1.2. T Cell Depletion with Reduced Intensity Conditioning

RIC conditioning with reduced doses of fludarabine, thiotepa, and melphalan [35] and a CD3/CD19 depleted graft using anti-CD3 monoclonal antibody (OKT-3) was tried in a limited number of patients. Because of the less myeloablative conditioning and lack of a megadose, several modifications were necessary to promote engraftment. OKT-3 was chosen over ATG as it was thought to spare incoming donor NK cells, which could aid in engraftment. Additionally, a newer method of T cell depletion was developed involving the use of microbeads coated with anti-CD3 and anti-CD19 to negatively deplete B- and T-lymphocytes, whilst CD34+ stem cells, CD34− progenitor cells, NK cells, dendritic cells, and other engraftment-facilitating cells were spared. The graft contained a median of 7.8 × 10^6^/kg CD34+ cells, 5 × 10^7^/kg CD56+ cells, and less than 2 × 10^4^/kg CD3+ cells.

This CD3/CD19 depleted haploidentical transplant was carried out on 61 adult patients with high-risk leukaemia. Rapid engraftment was observed with significant numbers of granulocytes and platelets present at 11 and 12 days after transplant, respectively. Overall TRM rates were comparable to the previous MA study [33], even in the older and higher-risk population used in this study. One limitation, however, was higher rates of grade II to IV acute and limited chronic GVHD (46% and 18%, resp.) when compared to the positively selected CD34+ transplants with cells probably as a result of higher doses of T cells being administered.

#### 4.1.3. Other Selective T Cell Depletion Methods

Other methods of selective T cell depletion are also being investigated in smaller clinical trials [36, 37]. For example, one study sought to first activate donor T cells in vitro by culturing the cells with host cells (known as a mixed lymphocyte culture system). Activated alloreactive T cells proceed to express the CD25 membrane protein (interleukin 2R*α*), allowing differentiation from nonalloreactive T cells. A CD25 immunotoxin was then used to selectively deplete these T cells whilst sparing those that assist in defence against infection [36].

Engraftment was achieved fully in 10 out of 16 paediatric patients in the study, with partial chimerism seen in five other patients. Early results indicated that there were no cases of GVHD above grade II, and importantly 12 patients showed signs of preserved immune responses.

Another study sought to use photodepletion to remove alloreactive T cells, which were found to selectively accumulate a photo-sensitizing compound known as 4,5-dibromorhodamine 123 (TH9402) [37]. Again, cells were stimulated in vitro and then selectively T cell depleted and infused into the patient. Of 24 patients who began the study, 11 patients survived for a median of 30 months. Incidence of grade III and IV GVHD was 13%, and six patients suffered relapse. Immune reconstitution, however, was still observed to be delayed.

#### 4.1.4. CD45RA Depletion

A novel strategy used to circumvent issues commonly encountered in T cell depleted transplants, (namely, delayed immune reconstitution and graft failure) is the specific depletion of naïve T cells and terminal effector cells. The CD45RA cell marker can be found on naïve T cells, fully matured cells that have never encountered antigens specific to their T cell receptor [38]. These cells remain alloreactive and proliferate upon activation. Studies have suggested that these CD45RA+ naïve T cells are largely responsible for posttransplant GVH reactions and that infusions of memory T cells, lacking CD45RA+ cells, do not induce GVHD reactions [39–41]. Further study has shown that selective depletion of CD45RA+ cells is possible in donor leukapheresis products [42].

A trial at St. Jude Children's Research Hospital in Memphis, USA, was conducted to investigate the feasibility of CD45RA+ depletion in haploidentical transplantation, for paediatric patients with high-risk malignancy [43]. Eight patients with relapsed or refractory solid tumours received two cell product infusions, CD3+ depleted and CD45RA+ depleted, respectively, from KIR2DL1 mismatched donors. Infusions met a minimum CD34+ dose of 2 × 10^6^ cells/kg and were below a maximum dose of 1 × 10^5^ CD3+ cells/kg. All eight patients engrafted neutrophils successfully within 14 days. Despite high-risk disease and the high likelihood of TRM, the regimen was well tolerated with no cases of acute GVHD. It is also important to note that GVHD prophylaxis consisted solely of a brief course of sirolimus.

#### 4.1.5. Treg/Tcon Haploidentical Transplants

Further attempts to manipulate transplants involve the use of combinations of regulatory T cells (Tregs) and conventional T cells (Tcons), working in tandem to provide substantial GVL effect whilst moderating the acute and chronic GVH effects. Thymus derived, naturally occurring Tregs are identified by CD4+ CD25+ FoxP3+ cell markers and are primarily involved in regulation of immune activity and maintenance of physiological self-tolerance [44, 45]. In animal models, these cells have been shown to suppress GVHD whilst maintaining the GVL effects of Tcons [46].

One study of 43 patients with high-risk acute leukaemia sought to investigate whether this Treg/Tcon coinfusion immunotherapy could replicate results seen in animal models [47]. Conditioning was MA and included TBI, thiotepa, and fludarabine, as well as either cyclophosphamide, alemtuzumab, or thymoglobulin. It is important to note that no GVHD prophylaxis was used. Overall results were encouraging, with a sustained engraftment rate of 95 percent and only 15 percent of patients developing acute GVHD (grades II to IV). The low rates of GVHD observed combined with the extremely low rates of relapse (cumulative incidence of 0.05) in a high-risk population suggest that the powerful Tcon derived GVL effect was maintained, despite Treg mediated GVHD suppression.

#### 4.1.6. Graft Selection and NK Cell Alloreactivity

Although it would be expected that the extensive depletion of T cells would lead to loss of the GVL effect and a subsequent increase in posttransplantation leukaemia relapse rate, this was not observed in the T cell depleted studies. A potential reason for this is the alloreactivity of transplanted donor-derived natural killer (NK) cells [48]. NK cells have been found to possess inhibitory receptors known as “killer cell immunoglobulin-like receptors” or KIRs, which recognise KIR ligands shared by self-HLA molecules. On ligand presentation, these KIRs become “licensed” to react to allogeneic targets that do not express self-HLA KIR ligands. In haplotype-mismatched transplantation, NK cells develop in the bone marrow surrounded by cells of donor haplotype and thus become alloreactive to recipient leukaemia cells that lack the donor HLA KIR ligand.

A study of 112 patients who received haploidentical transplants demonstrated that transplantation from NK alloreactive donors, those who possessed HLA class I KIR ligands which were absent in the recipient as well as alloreactive NK cell clones, had a significantly lower incidence of leukaemia relapse (3% versus 47%) when transplanted in remission. Furthermore, transplantation from NK alloreactive donors led to overall better event-free survival rates and reduced risk of relapse or death. Maternal donation also provided protection from leukaemia relapse, additional to any benefit gained from NK alloreactive donation [49]. This is thought to be a result of maternal exposure to foetal antigens during pregnancy, leading to maternal memory T cell tolerance to the paternal HLA haplotype present in the foetus. This information can be useful in choosing the best potential donor when several are made available, as might be the case with haploidentical transplantation.

### 4.2. T Cell Replete Transplantation

#### 4.2.1. High Dose Cyclophosphamide Posttransplantation

An alternative to complete T cell depletion is the selective depletion of T cells responsible for alloreactivity (leading to GVHD and graft rejection), whilst sparing the nonalloreactive T cell population which provides immune reconstitution and protection against infection (leading to a reduced TRM rate) [50, 51]. Cyclophosphamide (Cy) provides a unique way of achieving this. Cy has previously been used as part of a MA regimen, administered prior to transplantation to suppress the recipient immune system [52]. The effects of Cy are time dependent, and preclinical studies showed that when administered as a properly timed, high dose after transplantation (between 60 and 72 hours), it reduces incidence of both GVHD and graft rejection [53]. Cy is thought to affect both donor and recipient derived proliferating alloreactive T cells selectively, whilst sparing nonproliferating nonalloreactive T cells. It has also been observed to be less toxic to HSCs due to their high expression of its inactivating enzyme, aldehyde dehydrogenase (ALDH).

**Figure 1:**
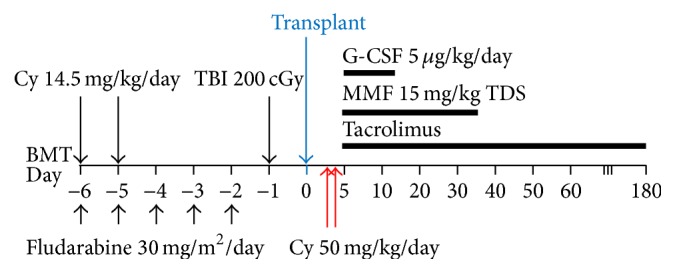
Conditioning regimen used for nonmyeloablative haploidentical transplantation, using high dose cyclophosphamide (Cy) posttransplant for in vitro T cell depletion. Pretransplant conditioning involved Cy, fludarabine, and TBI, with administration of high dose Cy on day 3 (or days 3 and 4) after transplantation. GVHD prophylaxis consisting of tacrolimus and MMF was initiated after Cy. BMT: bone marrow transplantation, Cy: cyclophosphamide, TBI: total body irradiation, G-CSF: granulocyte colony stimulating factor, and MMF: mycophenolate mofetil.

One study used a nonmyeloablative approach in order to reduce the likelihood of transplant-related mortality and also in the hope that, in case of graft rejection, autologous haematopoiesis could recommence [50]. Pretransplant conditioning involved Cy, fludarabine, and TBI, with administration of high dose Cy on day 3 (or days 3 and 4) after transplantation. GVHD prophylaxis consisting of tacrolimus and MMF was initiated after Cy. This regimen is illustrated in Figure 1. Whilst engraftment was sustained in a majority of patients (57 of 66), acute and chronic GVHD incidences were low (27% and 13% for grades II to IV) and NRM was relatively low (18%); incidence of relapse mortality was shown to be high (55%). This was thought to be partly due to a high-risk trial population and partly due to poor disease eradication by the nonmyeloablative conditioning used, in addition to the lack of GVL effect caused by depleting alloreactive T cells.

A similar procedure has also been carried out using peripheral blood stem cells (PBSCs) as opposed to bone marrow (BM) transplantation [54]. PBSCs are generally preferred in HLA-matched transplantation as they are easier to collect from donors, have higher yields of HSCs, and can lead to better short- and long-term survival. Due to their higher T cell content and increased risk of GVHD, they had previously been avoided in haploidentical transplants. Results have shown that RIC with posttransplant Cy can in fact lead to acceptable rates of GVHD, NRM, and relapse.

In another study, RIC with posttransplant Cy was also performed with conditioning consisting of thiotepa, busulfan, and fludarabine, or TBI plus fludarabine [55]. GVHD prophylaxis consisted of posttransplant Cy on days 3 and 5, ciclosporin, and MMF. Results showed low incidence of grade II to IV acute and chronic GVHD (12% and 10%, resp.) and low incidence of overall TRM (18%). Rate of relapse was also lower than that achieved by RIC in the previous study (26% in this study). Disease-free survival was also lower (68% for patients transplanted in remission and 37% for those transplanted in relapse).

One study has shown that haploidentical transplants using posttransplantation cyclophosphamide can achieve similar outcomes to MRD and MUD transplants [56]. Specifically, in 271 consecutive patients undergoing allogeneic transplantation for haematological malignancy, no significant difference in nonrelapse mortality, relapse, incidence of acute severe GVHD, and overall survival was found. This strategy has been lauded due to its relative success and has a major advantage over manipulated T cell depleted grafts as it can be performed in any centre performing HSCT and does not require specialist graft manipulation.

#### 4.2.2. Posttransplant Rapamycin

An alternative conditioning to promote immune tolerization by increasing circulating TRegs employed treosulfan, fludarabine, ATG, and rituximab for T and B cell depletion and rapamycin/mycophenolate mofetil [57, 58]. Rapamycin was chosen for its activity in the promotion of natural regulatory T cells (Tregs), shown in preclinical models, in contrast with calcineurin inhibitors such as ciclosporin. Tregs play an important role in the induction of transplant tolerance and the prevention of autoimmune reactions [59]. Preclinical models have shown that adoptive transfer of purified natural Tregs can prevent GVHD whilst leaving the desirable graft-versus-leukaemia effect unaffected. Rapamycin also inhibits effector T cell action and has direct antineoplastic activity against haematological malignancies such as acute leukaemia and thus could provide additional benefit.

Fifty-nine patients were transplanted, with conditioning consisting of treosulfan, fludarabine, and ATG and rituximab for T and B cell depletion. GVHD prophylaxis consists of rapamycin and MMF. Engraftment occurred in all patients and was reported to result in fast immune reconstitution. Incidences of acute GVHD (grades II to IV) and chronic GVHD were 29% and 20%, respectively, and incidences of TRM and relapse were 25% and 44%, respectively. High levels of circulating Tregs were observed, which were found to suppress effector T cells when placed in vitro.

#### 4.2.3. G-CSF Priming of Donor Bone Marrow

Another group of researchers sought to overcome the issues of T cell depletion via in vivo modulation of T cell function in the recipient and the donor [60]. The protocol they developed was termed GIAC, representing the four significant protocol adjustments: donor treatment with granulocyte colony stimulating factor (G-CSF), intensified immunologic suppression, infusion of ATG for GVHD prophylaxis, and use of a combination of PBSC and BM cells. Donors were treated with recombinant G-CSF for 5 to 6 consecutive days, with BM harvest occurring on the 4th day and PBSC collection occurring on the 5th day. Additional cells were collected on day 6 if the previous collections were insufficient. Cells were then infused into the recipient, unmanipulated, on the same day. The recipient was conditioned using cytosine arabinoside, busulfan, Cy, semustine, and ATG and treated after transplant with ciclosporin, MMF, and methotrexate as GVHD prophylaxis.

Two hundred and fifty patients with acute leukaemia underwent haploidentical transplantation using this regimen. Of these, 249 had sustained engraftment, with incidence of grade II to IV acute and chronic GVHD being 45.8% and 53.9%, respectively. Notably, the rates of GVHD were similar to those of HLA-identical allogeneic HSCT, in spite of the lack of extensive in vitro depletion of T cells. The mechanism for this is thought to be a combination of the effects of G-CSF priming of donor cells, in maintaining T cell hyporesponsiveness and encouraging the development of tolerant Th2 cells, as well as the in vivo effects of GVHD prophylaxis and ATG in the conditioning. Furthermore, probability of leukaemia-free survival for standard and high-risk AML was 70.7% and 55.9% and for ALL was 59.7% and 24.8%, which is comparable with previous studies.

Subsequently, another group of researchers has modified this protocol slightly, only using a G-CSF primed BM graft as opposed to combining it with HSCs [61]. Patients in this protocol either were conditioned with myeloablation using cytarabine and cyclophosphamide plus TBI, treosulfan, or busulfan or underwent RIC using fludarabine and melphalan. Extensive GVHD prophylaxis was also used via a combination of five drugs with differing mechanisms, ATG, ciclosporin, methotrexate (MTX), MMF, and basiliximab, an anti-CD25 monoclonal antibody.

80 patients with high-risk haematologic malignancies were transplanted using this protocol. An engraftment rate of 93% was achieved, with incidence of grade II to IV acute and chronic GVHD being 24% and 6%, respectively. Probability of overall survival at three years was 54% for standard risk patients (treated in first or second complete remission) and 33% for high-risk patients (treated in third or later remission, or in active disease).

The largest study done to date using the GIAC protocol involved 1210 consecutive patients transplanted between May 2002 and February 2013 at Peking University Institute of Hematology in China [62]. The study found that under this standardised protocol the degree of HLA mismatch did not significantly correlate with transplant outcomes. Rather, donor characteristics such as age, gender, family relationship, and microchimerism effects (expanded below) were more prognostic of favourable outcomes. Despite the large study number, experience and further data are limited outside of this institution.

#### 4.2.4. Fetomaternal Microchimerism

**Figure 2:**
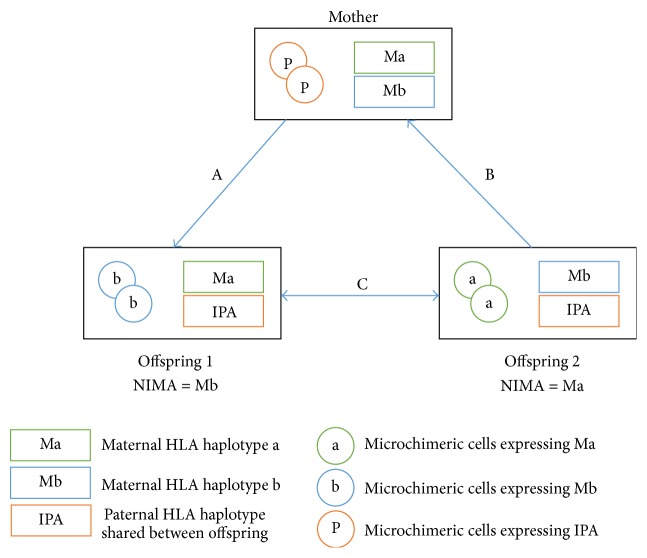
Illustration of three types of NIMA-complementary HLA haploidentical stem cell transplants: A, B, and C. Transplantation from mother to offspring (A) causes a graft versus host (GVH) reaction against the inherited paternal antigen (IPA) and a host versus graft (HVG) reaction against the NIMA of offspring 1 (Mb). Transplantation from offspring to mother (B) causes a GVH reaction against the NIMA of offspring 2 (Ma) and HVG reaction against the inherited paternal antigen (IPA). Transplantation between NIMA-mismatched siblings with a shared IPA (C) involves bidirectional mismatch for the NIMA and bidirectional GVH/HVG reactions. Adapted from Ichinohe et al. [63].

Ichinohe and colleagues hypothesised that the rates of GVHD and graft rejection could be attenuated by transplanting T cell replete bone marrow or peripheral blood stem cells from mismatched family donors, such as sibling or children donors who were microchimeric for the noninherited maternal antigen (NIMA), or a mother who was microchimeric for the inherited paternal antigen (IPA) [63]. Prior exposure to either ingested or circulating mismatched antigen would have tolerised these donors; hence, T replete transplants with relatively shorter duration of immunosuppression would be possible, offsetting the risks of poor immune reconstitution. A summary of this is illustrated in Figure 2.

Ichinohe et al. reported the multicentre outcomes of 35 such transplants for haematological malignancies (AML 12, ALL 11, CML 7, and lymphoma 5) [63]. The transplants were T cell replete (30 patients received GCSF mobilised PBSC harvests, four patients bone marrow harvests, and one patient a combination of both), predominantly myeloablative (21 patients/60%) transplant with tacrolimus/methotrexate GVHD prophylaxis in 63%. Posttransplant GCSF was administered in 28 patients. NIMA and IPA haplotypes were deduced based on tissue typing family members from 2 or 3 generations. The presence of recipient specific long-term microchimerism was confirmed by IPA or NIMA specific nested PCR with sequence specific primers. Fifteen donors were IPA mismatched (maternal donor) and 20 were NIMA mismatched (sibling or offspring). Engraftment occurred in 33 evaluable patients at a median of 14 days. The cumulative incidence of grades II–IV aGVHD was 56% (95% CI 38–71%) and III-IV 22% (95% CI 10–37%). The incidence of grades III-IV aGVHD in the NIMA mismatched group was 10% (95% CI 2–26%) and significantly lower than the IPA mismatched group 38% (95% CI 15%–60%). The incidence of cGVHD was 83%, six patients had limited cGVHD, and 13 had extensive cGVHD. Extensive cGVHD occurred in 4/9 patients (44%) in the IPA targeted group and 9/15 (66%) patients of the NIMA targeted group. The estimated probability of survival was 38% (95% CI 17–60%) for the whole cohort.

These results were encouraging but highlighted that in some individuals severe GVHD occurred despite fetomaternal chimerism, suggesting that whilst this was a relative indicator of hyporesponsiveness, it was not absolute. In particular, severe GVHD in the NIMA-complementary mismatch was associated with IPA mismatch in the GVH direction. Studies by Cai et al. [64] elucidated that the tolerance associated with long-term fetomaternal chimerism is determined by the balance between regulatory T cells (Tregs) and T effector cells (Teff) specific for IPAs or NIMAs. Tests to evaluate Treg versus Teff balance such as allopeptide-specific tetramer staining or trans vivo delayed type hypersensitivity assays (injection of cryopreserved human PBMCs into the footpad of a CB17 SCID mouse footpad with the relevant antigenic peptide and controlling or neutralising antibody) are available and may help in further clarifying the role of fetomaternal microchimerism in allospecific tolerance after mismatched haploidentical transplants.

#### 4.2.5. The Role of HLA Matching in Donor Selection

The degree of HLA mismatching often predicts increased GVHD and lower relapse rates in the allogeneic transplant setting. In a retrospective analysis of 185 patients with haematological malignancies transplanted with the RIC posttransplant Cy protocol, no correlation was found between the number of HLA mismatches, the risk of acute grade II–IV GVHD, graft failure, and event-free survival [83]. The Peking group studied 1210 consecutive haploidentical transplants, treated on the GIAC protocol to identify the optimal donor, and found that HLA mismatching did not correlate with outcomes. Instead, lower NRM and better survival were evident with younger donors and male donors. Fathers demonstrated lower NRM and aGVHD than mothers, and children donors demonstrated lower aGVHD than siblings. For siblings with NIMA mismatches, the rates of aGVHD were lower compared with those with noninherited paternal antigen (NIPA) mismatches [62]. An EBMT registry study of 173 adults with AML and 93 with ALL transplanted with the T deplete megadose, CD34 approach confirmed that the degree of HAL mismatch did not correlate with outcomes in this setting [84].

## 5. Haploidentical Transplants for Haematological Malignancies

To date, a number of studies have been completed evaluating haploidentical transplants against several outcomes including rate of engraftment, relapse, and acute and chronic GVHD, as well as event-free survival and overall survival. In some cases, these transplants have been compared contemporaneously with MRD, MUD, mismatched unrelated donor, and cord blood transplants.  Table 1 provides a summary of these studies, which are described in detail in the following.

### 5.1. Myelodysplastic Syndrome (MDS) and Acute Myeloid Leukaemia (AML)

Single centre analyses of haploidentical transplant for MDS/AML report encouraging data for engraftment, acute and chronic GVHD, overall survival, and event-free survivals that are comparable to matched unrelated donor transplants [65–67]. Two studies focused on patients with acute leukaemia including AML [69, 70], and twelve other trials included one or more patients with AML or MDS among others [54, 56, 73–82].

Amongst these, a large CIBMTR retrospective study compared 192 haploidentical (predominantly bone marrow graft and posttransplant Cy) with 1982 8/8 matched unrelated donor (predominantly peripheral blood grafts) transplants for AML [65]. The study demonstrated that, for MA transplants, neutrophil engraftment was at 90% for haploidentical transplants but 97% for MUD, whereas for RIC haploidentical transplant and MUD it was 93% and 96%, respectively. Reassuringly, aGVHD, grades II to IV, at 3 months for MA haploidentical transplant was low at 16% with this being doubled at 33% with MUD transplants (*p* < 0.001). Similarly, cGVHD at three years was 30% in MA haploidentical transplant and 53% for MUD (*p* < 0.001). This pattern is replicated with RIC transplants with aGVHD grades II to IV being 19% compared to 28% (*p* = 0.05) with MUD, and cGVHD at 3 years being 34% versus 52% (*p* = 0.002). These translate into comparable probabilities of overall survival (OS) of 45% (95% CI 36–54) and 50% (95% CI 47–53%) with haploidentical transplant and MUD MA transplants and 46% (95% CI 35–56) and 44% (95% CI 40–47) for haploidentical transplant and MUD RIC transplants, respectively. Although this retrospective analysis is not powered to detect small differences, these data are reassuring that mismatched family donors afford equivalent results.

A Chinese study also compared outcomes of patients with AML transplanted with a T replete low dose ATG conditioned transplant [85]. Of these, 90 patients had matched sibling donors (MSD), 116 had unrelated donors, and 99 haploidentical related donors (HRD). With this conditioning, the rates of aGVHD grades II–IV were 42.4% and grades III-IV were 17.2%, which resulted in a higher nonrelapse mortality (NRM) of 30.5%. This seems however to lead to a lower risk of relapse at 5 years in HRD of 15.4% in comparison with 28.2% with URD and 49.9% with MSD (*p* = 0.002). The 5-year disease-free survival (DFS) for those undergoing transplant with the three donors was 63.6% for MSD, 58.4% for URD, and 58.3% for HRD. This group then prospectively compared consecutive transplants between 2010 and 2013 for AML in CR1 with intermediate or high-risk disease who had either MSD (219 patients) or HRD (231 patients) transplanted with very similar conditioning, including ATG for haploidentical transplants and Cy/MTX for MSD [67]. Whilst the haploidentical group tended to be younger, the results for both groups were similar in terms of aGVHD and cGVHD with NRM in HRD being 10% (similar to that seen with posttransplant Cy, 7% at 1 year). These GVHD rates were also comparable to those with posttransplant Cy. Additional MRD monitoring and preemptive DLI or therapeutic DLI resulted in low relapse rates of 15% with both donors. The 3-year probabilities of leukaemia-free survival were 76% (95% CI 64–87) and 80% (95% CI 70–91) for HRD and MSD, respectively. Three-year probabilities of OS were 79% (95% CI 73–85) and 82% (95% CI 76–88) for HRD and MSD, respectively. Features of the underlying leukaemia including cytogenetics and white cell count >50 × 10^9^/L at presentation were independent predictors of outcome on multivariate analysis.

As patients with AML and MDS are older, the comparison of transplant outcomes by donor type in the older recipient is relevant. Blaise et al. [74] compared outcomes of 31 patients older than 55 years (predominantly MDS/AML) transplanted with RIC PB Cy haploidentical transplants with MSD and MUD conditioned with ATG and cyclosporine (CsA) ± MMF in the same age group. Whilst GVHD rates were comparable (CI 23% Haplo, 21% MSD) for related donors, it was higher (CI 44%) for MUD. No patient with HRD developed cGVHD whereas 16% of MSD and 14% of MUD developed this. Importantly, the cumulative incidence of relapse (CIR) was similar in all the groups but NRM after an MUD was threefold higher compared to a related donor. Thus, 2-year OS was 70%, progression-free survival (PFS) was 67%, and severe cGVHD-free survival was 67% after HRD transplant. Similar outcomes were seen with MSD (78%, 64%, and 51%) whereas the results in this small cohort for URD transplants were 51% (*p* = 0.08), 38% (*p* = 0.02), and 31% (*p* = 0.007), respectively.

These reports build a case for HRD to be considered where a MSD is unavailable. They may be preferred over MUD, in the older patient particularly with posttransplant Cy due to reduced side effects of transplant and in all recipients where an 8/8 MUD is not available.

### 5.2. Acute Lymphoblastic Leukaemia (ALL)

There are fewer reports of the efficacy of modern platforms of T cell replete haploidentical stem cell transplantation for ALL. A report from southwest China studied 82 patients with Philadelphia chromosome positive ALL, transplanted with MSD in 35 patients and HRD in 47 patients [68]. The conditioning regimen consisted of 9–10.5 Gy TBI and cyclophosphamide 60 mg/kg for two days in the MSD group, with the addition of 6 g/m^2^ ATG IV for 3 days in the haploidentical transplant group and reduction of Cy to 45 mg/kg/day for 2 days. GVHD prophylaxis in the MSD consisted of CsA, MTX, and MMF to day 30 whereas the haploidentical transplant group received additional ATG and MMF to 90 days. Most patients were transplanted in molecular remission but* bcr-abl* was detectable at circa 2% in 7 MSD and 10 HRD before transplant. Imatinib was commenced after transplant when* bcr-abl* was detected molecularly. The cumulative incidence of GVHD both acute (51% HRD versus 26% MSD) and chronic (49% HRD versus 26% MSD) was higher in the HRD and appeared to exert a GVL effect with a CIR at 9.1% being lower than that for MSD 19.1%, HR 0.413 (95% CI 0.178–0.958). Transplantation in CR > 1 was predictive of relapse. The study provides encouraging data for a HRD in the absence of MUD.

Two further studies considered acute leukaemia including ALL [69, 70]. In particular, EBMT registry data compared the outcomes of haploidentical transplant with UCBT for 918 acute myeloid leukaemia patients (HRD 360 patients and UCBT 558 patients) and 528 acute lymphoblastic leukaemia patients (HRD 158 patients and UCBT 370 patients). For ALL, although the HRD had significantly more patients with advanced stage disease (48% versus 34%, *p* = 0.02) and poor risk cytogenetics (26% versus 14%, *p* = 0.03), OS and DFS were similar to both graft sources whereas the incidence of cGVHD was lower with UCBT. Nonengraftment was higher with UCBT but did not translate into a higher NRM. Transplant conditioning regimens were heterogeneous and cGVHD rates were higher for HRD than those reported from single centre studies. The deleterious effect of RIC on relapse and advanced disease on transplant outcomes was seen in LFS, NRM, and relapse (*p* < 0.001). Notwithstanding these criticisms, results from this large dataset suggest that both sources of stem cells would be appropriate in the absence of a suitable matched donor. Further twelve studies with mixed patient groups all included patients with ALL among others [54, 56, 73–82].

### 5.3. Hodgkin's Lymphoma (HL)

Burroughs et al. [71] transplanted patients with Hodgkin's lymphoma with relapsed or refractory disease, treated with a median of five chemotherapy regimens, with MRD (38 patients), MUD (24 patients), or HRD (28 patients with the RIC BM, posttransplant Cy). Whilst OS was similar at 2 years (53% MRD, 58% MUD, and 58% HRD), relapse was less frequent with HRD 40% versus 63% with MUD and 56% with MRD resulting in a PFS of 51% with HRD compared to 29% with MUD and 23% with MRD. These were the initial set of data where a HRD transplant resulted in outcomes superior to conventional donor, paving the way for further such comparisons. A number of mixed studies also included patients with HL [54, 56, 73–75, 81, 82] or unspecified lymphoma [80].

A recent study published by Kanate et al. [72] compared 917 adult patients with both Hodgkin and non-Hodgkin lymphoma, undergoing either haploidentical (*n* = 185) or MUD (*n* = 732) transplants. The MUD transplant group was further divided into conditioning regimens with additional ATG as GVHD prophylaxis and those without. The study found the haploidentical group to be at significantly reduced risk of severe acute GVHD (grades III and IV) compared to ATG and non-ATG groups (8% versus 12% and 17%, resp., *p* = 0.01 and 0.001). There was also significantly reduced risk of chronic GVHD (13% versus 51% and 33%, *p* < 0.0001). The study also demonstrated no significant differences in OS between both MUD groups and the haploidentical group and showed no overall difference in NRM, relapse, and PFS. These results provide further evidence that haploidentical transplants may be an acceptable option for lymphoma patients lacking an HLA-identical donor and can be safely chosen over MUD donors without compromising survival outcomes.

### 5.4. Non-Hodgkin's Lymphoma (NHL) and Chronic Lymphocytic Leukaemia (CLL)

Ten of the twelve studies with mixed patient groups included patients with NHL [54, 56, 73–78, 81, 82] whilst five of these also included patients with CLL [54, 56, 73–75]. None of these studies reported outcomes for these diseases separately.

In one of the bigger comparative studies, Bashey et al. [56] retrospectively looked at 271 consecutive transplant patients at their centre in Atlanta, USA. Fifty-three patients underwent haploidentical transplants, 117 underwent MRD, and 101 underwent MUD transplants. Several different conditioning regimens were used for the MRD and MUD groups. In the haploidentical group, 35 patients underwent nonmyeloablative conditioning with fludarabine (30 mg/m^2^), 2 Gy TBI, and Cy (14.5 mg/kg before transplant and 50 mg/kg after transplant), whilst 18 patients underwent myeloablative conditioning involving fludarabine (25 mg/m^2^), busulfan (110–130 mg/m^2^), and Cy (14.5 mg/kg before transplant and 50 mg/kg after transplant). Tacrolimus, MMF, and G-CSF were also used in all patients. Rates of relapse, NRM, aGVHD, cGVHD, 2-year OS, and DFS for MRD, MUD, and haploidentical transplants were nonsignificant across all groups.

In a later study, Bashey et al. [73] retrospectively analysed further 475 consecutive transplant patients at their centre, with a longer follow-up period than the previous study. Transplants were either MRD (181 patients), MUD (178 patients), or haploidentical (116 patients) with a heterogeneous mixture of conditions including 70 NHL and 26 CLL patients as well as 170 AML and 62 ALL among others. Conditioning for MRD and MUD groups varied whilst a posttransplant Cy regimen was used for haploidentical transplants. At 2-year follow-up, OS was comparable in haploidentical and MUD groups (57% versus 59%, resp., *p* > 0.05), as well as DFS (54% versus 50%), NRM (17% versus 16%), and acute GVHD (41% versus 48%). Rates for moderate to severe cGVHD were observed to be lower in the haploidentical group (31% versus 47%, *p* = 0.004), and haploidentical transplants were less likely to receive systemic immunosuppressive treatment (19% versus 42%, *p* = 0.007). Results for MRD were significantly superior for OS and aGVHD only. The study went on to suggest that haploidentical transplants are an appropriate alternative in patients lacking a fully matched related donor. Fully HLA-matched unrelated donors (10/10 HLA alleles) have been previously shown to be superior to mismatched unrelated donor transplants (>1 mismatched allele); hence, it was suggested that haploidentical transplants may in fact be a more appropriate alternative than mismatched transplants.

Raiola et al. [81] conducted a study of 459 consecutive patients with a variety of malignancies including 232 patients with acute leukaemia (AML/ALL) and 59 patients with lymphoma (HL/NHL). Transplants were categorised as being either a matched sibling donor (MSD), MUD, mismatched unrelated donor (mmUD), unrelated cord blood donor (UCD), or haploidentical donor. Haploidentical transplants were either myeloablative using thiotepa, busulfan, and fludarabine or nonmyeloablative using TBI and fludarabine. Regimens also included posttransplant Cy, CsA, and MMF. Acute GVHD was significantly lower in the haploidentical group (14%) compared to the MSD and mmUD groups (31% and 42%, *p* < 0.001), and there was a similar but not statistically significant pattern for chronic GVHD (*p* = 0.053). Haploidentical transplant recipients had the highest 4-year OS (52%), which was nonsignificant across all groups (*p* = 0.10) but comparable with MSD transplants in multivariate analysis (45%, *p* = 0.80). Again, results suggested that haploidentical transplants could be a valid option, comparable to MUD transplants, in the absence of an HLA-identical related donor.

Castagna et al. [75] sought to compare the use of PBSC and BM in haploidentical patients. Sixty-nine consecutive patients (46 BM and 23 PBSC) were analysed in this retrospective study, including 29 patients with HL, 24 with NHL, and 4 with CLL among others. A nonmyeloablative regimen with posttransplant Cy was used in both groups of patients. Results showed similar rates of acute and chronic GVHD in PBSC and BM groups (33% versus 25%, *p* = 0.43, and 13% versus 13%, *p* = 0.21) as well as similar NRM (12% versus 22%, *p* = 0.96), suggesting that PBSC transplants using this regimen were not inferior to BM transplants for this regimen used. A major limitation of this study was the small number of patients included; the limited data nonetheless suggest that PBSCs are a valid option in haploidentical transplants compared to BM transplants.

### 5.5. Multiple Myeloma (MM)

Few haploidentical transplants for multiple myeloma with posttransplant Cy have been included in studies [56, 73–75]; however, outcomes are not reported separately. A single series reported on 10 patients transplanted with the RIC PBSC posttransplant Cy haploidentical transplant with TBI doses between 2 Gy and 4 Gy [86]. The median age of patients was 53 years (range 28–61 years) who had relapsed or were refractory after autograft. Fludarabine 30 mg/m^2^ was added to the protocol if CD4 counts before transplant were greater than 200 × 10^9^/L. Bone marrow graft was used in six patients and PBSC in four patients. Engraftment kinetics of neutrophils and platelets were similar to those reported from other posttransplant Cy RIC studies. Median neutrophil engraftment occurred by day 18 and platelet engraftment occurred by day 17. The median OS was 443 days; OS at 1 year was 61.7% and at 2 years was 46.3%. Causes of death included sepsis in one patient and disease progression in two patients. Grade II–IV aGVHD occurred in 8/10 patients and five developed cGVHD. Relapse occurred in five patients with a median time to progression of 7.8 months: of these, two patients were salvaged with chemotherapy and DLI. More data are needed.

### 5.6. Myeloproliferative Neoplasms/Disorders (MPD)

Raiola et al. [80] transplanted 50 patients with a variety of high-risk haematologic malignancies, which included four patients with primary myelofibrosis and one patient with MPD. Myeloablative conditioning consisted of either thiotepa (5 mg/kg/day ×2), fludarabine (50 mg/m^2^/day ×3), or busulfan (3.2 mg/kg/day ×3). The F-TBI regime consisted of 3.3 Gy TBI/day ×3 and fludarabine (30 mg/m^2^/day ×4). Posttransplantation Cy, CsA, and MMF were also used. Neutrophil engraftment occurred in 90% of patients in a median of 18 days. Incidence of acute GVHD was 12% and limited to grades II to III. Ten patients (26%) developed chronic GVHD of which six were mild. Overall incidence of relapse, TRM, and OS were 26%, 18%, and 68%, respectively. Results for patients with myelofibrosis were not reported individually.

One of the largest studies involving patients with MPD was by Raiola et al. [81] described previously. Studies by Raj et al. [54] and Solomon et al. [82] both included three patients each with CML; however, results were not reported separately. A number of other studies also included patients with CML [56, 73, 74, 79, 80] or unspecified myeloproliferative disorder (MPD) [73, 74, 76, 80, 81].

## 6. Conclusion and Future of Haploidentical Transplants

Undoubtedly, the techniques now used in haploidentical transplantation have had a positive impact on several outcome measures, namely, overall survival, disease-free progression, and survival free from acute or chronic GVHD. The studies described above are just a selection of cases where haploidentical transplants can feasibly be conducted on patients without an available HLA-matched donor. Recent retrospective analyses are beginning to assert that the paradigm for donor selection is changing, with haploidentical donors vying with MUD. Early data from China would suggest that these donors perform as well as MRD but this needs to be validated in further studies.

The ease of posttransplant Cy has led to its adoption across the world with data from EBMT showing an increasing trend for haploidentical transplants as opposed to stagnant levels of UCB transplantation [14]. With disease risk stratification, the role of the underlying disease on subsequent relapse has become clearer. It is increasingly clear that for patients with disease risk index, low/intermediate disease outcomes with haploidentical transplants are encouraging due to intrinsically lower relapse rates and low rates of acute GVHD and cGVHD. However, strategies to overcome high-risk disease with all types of donors remain to be identified. In this respect, engineered grafts with NK alloreactive donors may come to the fore.

It is important to note, however, that the majority of clinical data currently gathered for haploidentical transplants come from nonrandomised trials with retrospective comparison. Because of this, it is difficult to interpret data and compare and declare definitively whether one method is superior to another. Bearing this in mind, current recommendations are based on the expertise of the centre performing the transplantation and the facilities available, for example, for accommodating manipulation of grafts.

More studies, particularly randomised trials, are most certainly necessary before haploidentical transplants can be more readily used and their numerous benefits can be substantiated. Particularly, comparisons between donor sources (such as MRD, MUD, umbilical cord, and haploidentical donor) would be useful, as well as comparisons between various conditioning regimens and graft manipulation (or lack thereof). Studies can also be improved by recruiting more patients, focusing on specific conditions to remove confounding by disease variables and following-up patients for longer. Based on these, it may then be possible for haploidentical transplantation to fulfil its potential in providing substantial benefit to the field of allogeneic haematopoietic stem cell transplantation.

## Figures and Tables

**Table 1:** Summary of studies evaluating use of haploidentical transplantation, along with reported outcomes.

Study	Indications for transplant	HSC manipulation	Study group	*N*	Conditioning	HSC source	Engraft rate (%)	aGVHD II–IV (%)	cGVHD (%)	Relapse (%)	PFS/DFS/EFS (%)	OS (%)
[65]	2174 AML	Unmanipulated (T cell replete)	Haplo	192	104 myeloablative CNI, MMF, and PTCy	85 BM, 19 PB	90^*∗*^	16^*∗*^	30^*∗*^	44	24^*∗*^	3 yr OS 45
					88 RIC CNI, MMF, and PTCy	77 BM, 11 PB	93	19	34^*∗*^	58^*∗*^	18	3 yr OS 46
			MUD (8/8)	1982	1245 myeloablative CNI + MMF/MTX	231 BM, 1014 PB	97^*∗*^	33^*∗*^	53^*∗*^	39	12^*∗*^	3 yr OS 50
					737 RIC CNI + MMF/MTX	80 BM, 657 PB	96	28	52^*∗*^	42^*∗*^	10	3 yr OS 44

[66]	227 AML/MDS	Unmanipulated (T cell replete)	MRD	87	Fludarabine, melphalan, tacrolimus, and mini-MTX	2 BM, 23 PB	99	31	43	28	36	56
			MUD	108	Fludarabine, melphalan, tacrolimus, mini-MTX, and ATG	10 BM, 16 PB	96	29	30	23	27	
			Haplo	32	Fludarabine, melphalan, thiotepa, PTCy, tacrolimus, and MMF	18 BM, 1 PB	97	29	19	33	30	66

[67]	450 AML	Unmanipulated (T cell replete)	Haplo	231	Cytarabine, busulfan, Cy, Me-CCNU, and ATG; CsA, MMF, and MTX	Combined	100	36^*∗*^	42^*∗*^	15	74	79
			MRD (Sib)	219	Hydroxycarbamide, cytarabine, busulfan, Cy, Me-CCNU, and ATG; CsA, MMF, and MTX	14 BM, 81 PB, 124 combined	100	13^*∗*^	15^*∗*^	15	78	82

[68]	82 ALL	Unmanipulated (T cell replete)	MRD/MUD	35	TBI, Cy, MMF, CsA, and MTX ± imatinib	Not specified	^*∗∗∗*^	26^*∗*^	26^*∗*^	45^*∗*^	46^*∗*^	63
			Haplo	47	Ara-C, Cy, ATG, MMF, CsA, and MTX ± imatinib			51^*∗*^	49^*∗*^	19^*∗*^	60^*∗*^	64

[69]	918 AML	Unmanipulated (T cell replete)	CB	558	Various (276 MAC, 280 RIC)	558 cord	84^*∗*^	31	24		38	
			Haplo	360	Various (219 MAC, 141 RIC)	171 BM, 175 PB, 14 combined	91^*∗*^	27	29	(NS)^*∗∗∗*^	32	^*∗∗∗*^
	528 ALL	Unmanipulated (T cell replete)	CB	370	Various (261 MAC, 108 RIC)	370 cord	80^*∗*^	31	25		28	
			Haplo	158	Various (111 MAC, 43 RIC)	65 BM, 78 PB, 15 combined	94^*∗*^	33	31		34	

[70]	7874 AML, 2805 ALL	T cell depleted	MRD	297	Various	1585 BM, 8174 PB, 56 combined		24	30^*∗*^	31	48	
		Unmanipulated (T cell replete)		9518	Various		^*∗∗∗*^		37^*∗*^	32	52	^*∗∗∗*^
		T cell depleted	Haplo	268	Various	284 BM, 544 PB, 36 combined		25	17^*∗*^	25	21	
		Unmanipulated (T cell replete)		596	Various				27^*∗*^	40	30	

[71]	90 HL	Unmanipulated (T cell replete)	MRD	38	Various: TBI ± fludarabine, MMF, or CsA + tacrolimus	38 PBSC	95	50	50	56	23	53
			MUD/mmUD	24		24 PBSC	99	50	63	63	29	58
			Haplo	28	Nonmyeloablative: Cy, fludarabine, TBI, PTCy, tacrolimus, and MMF	28 BM	100	43	35	40^*∗*^	51^*∗*^	58

[72]	718 NHL, 199 HL	Unmanipulated (T cell replete)	MUD	241	Various (fludarabine + busulfan/Cy/melphalan ± TBI) + ATG	26 NM, 279 PBSC	97	49	33^*∗*^	36	38^*∗*^	50^*∗*^
				491	Various (fludarabine + busulfan/Cy/melphalan ± TBI) without ATG	31 BM, 460 PBSC	97	40	51^*∗*^	28	49^*∗*^	62^*∗*^
			Haplo	185	RIC (fludarabine, Cy, TBI, and PTCy)	172 BM, 13 PBSC	94	27	13^*∗*^	36	47^*∗*^	60^*∗*^

[55]	26 HL	Unmanipulated (T cell replete)	Haplo	26	(RIC) Cy, fludarabine, low dose TBI, PTCy, tacrolimus/CsA, and MMF	26 BM	96	24	9	31	63	77

[56]	91 AML, 44 NHL, 41 ALL, 26 CML/MPD, 22 MDS, 17 HL, 15 CLL, 13 MM, and 2 others	Unmanipulated (T cell replete)	MRD	117	Various	7 BM, 108 PB, 2 combined	98	8	54^*∗*^	34	53	76
			MUD	101	Various	6 BM, 92 PB	98	11	54^*∗*^	34	52	67
			Haplo	53	35 nonmyeloablative: fludarabine, TBI, Cy, PTCy, tacrolimus, and MMF. 18 myeloablative: fludarabine, busulfan, Cy, PTCy, tacrolimus, and MMF	32 BM, 21 PB	98	11	38^*∗*^	33	60	64

[73]	170 AML, 62 ALL, 70 NHL, 53 MDS, 29 HL, 29 CML, 26 CLL, 21 MPD, and 15 MM	Unmanipulated (T cell replete)	MRD	181	Various. Tacrolimus and MTX for GVHD prophylaxis	2 BM, 179 PBSC	98	21^*∗*^	58	30	56	72^*∗*^
			MUD	178	Various. Tacrolimus and MTX for GVHD prophylaxis	32 BM, 146 PBSC	98	48	62	34	50	59
			Haplo	116	Nonmyeloablative (fludarabine, TBI, Cy, and PTCy) or myeloablative (fludarabine, busulfan, Cy, PTCy or fludarabine, TBI, and PTCy)	64 BM, 52 PBSC	97	41	38^*∗*^	29	54	57

[74]	42 AML, 30 NHL, 22 MDS, 17 MM, 10 ALL, 10 CLL, 4 MPD, 3 HL, and 3 CML	Unmanipulated (T cell replete)	MRD	47	RIC (fludarabine, busulfan, ATG, CsA, and MMF)	47 PB	100	21	35^*∗*^	25	64	78
			MUD/MMUD	63	RIC (fludarabine, busulfan, ATG, CsA, and MMF)	3 BM, 60 PB	100	44	24	31	38^*∗*^	51
			Haplo	31	Initially nonmyeloablative (Cy, fludarabine, and TBI) increased if necessary to RIC (Cy/thiotepa, fludarabine, and busulfan) + PTCy, CsA, and MMF	4 BM, 27 PB	97	23	13^*∗*^	23	67^*∗*^	70

[75]	29 HL, 24 NHL, 4 CLL, 4 AML/MDS, 4 MM, and 2 ALL	Unmanipulated (T cell replete)	Haplo BM	46	(RIC) Cy, fludarabine, low dose TBI, PTCy, tacrolimus/CsA, and MMF	46 BM	87	25	13	^*∗∗∗*^	62^*∗∗*^	68^*∗∗*^
			Haplo PB	23		23 PB	95	33	13	^*∗∗∗*^		

[76]	38 AML, 20 ALL, 11 NHL, 7 MDS/MPD, and 1 SAA	Fixed CD3 DLI, positive CD34 selection	MRD	27	TBI, Cy, tacrolimus, and MMF	27 DLI	100	8^*∗*^	12	27	70	71
			Haplo	50	TBI, Cy, tacrolimus, and MMF	50 DLI	96	4077^*∗*^	19	21	68	70

[77]	16 AML, 4 ALL, 3 NHL, 2 MDS, 1 SAA, and 1 other	Fixed CD3 DLI w/OKT3	Haplo	27	TBI, Cy, tacrolimus, and MMF	27 PB	85	59	16	32	^*∗∗∗*^	48

[78]	15 AML, 10 ALL, 2 NHL, and 1 MDS	Fixed CD3 DLI, positive CD34 selection	Haplo	28	TBI, Cy, tacrolimus, and MMF	28 PB	100	39	22	^*∗∗∗*^	2 yr DFS 74	2 yr OS 77

[79]	15 AML, 2 ALL, 2 MDS, and 1 CML	Unmanipulated (T cell replete)	Haplo 1 × PTCy	9	Fludarabine, cytarabine, ATG, busulfan/melphalan, and PTCy	9 PB	95	56	10	^*∗∗∗*^	35	44^*∗*^
			Haplo 2 × PTCy	11	Fludarabine, cytarabine, ATG, busulfan/melphalan, and PTCy × 2	11 PB		64				64^*∗*^

[80]	25 AML, 12 ALL, 5 HL/NHL, 4 MF, 3 CML, and 1 MPD	Unmanipulated (T cell replete)	Haplo	50	Thiotepa, busulfan, and fludarabine; PTCy, CsA, and MMF; or TBI, fludarabine, PTCy, CsA, and MMF	50 BM	90	12	26	26	51	62

[81]	232 AML/ALL, 59 HL/NHL, 74 MPD, 81 MDS, and 13 others	Unmanipulated (T cell replete)	MRD (Sib)	176	Various myeloablative or RIC. GvHD prophylaxis CsA, MTX	156 BM, 20 PB	^*∗∗∗*^	31^*∗*^	29	40	32	45
			MUD	43	Various myeloablative or RIC. GvHD prophylaxis CsA, MTX, and ATG	26 BM, 17 PB		21^*∗*^	22	23	36	43
			MMUD	43	Various myeloablative or RIC. GvHD prophylaxis CsA, MTX, and ATG	28 BM, 15 PB		42^*∗*^	19	30	34	40
			CB	105	Various myeloablative or RIC. GvHD prophylaxis CsA, MMF, and ATG	105 cord		19^*∗*^	23	30	33	34
			Haplo	92	Thiotepa, busulfan, and fludarabine; PTCy, CsA, and MMF; or TBI, fludarabine, PTCy, CsA, and MMF	92 BM		14^*∗*^	15	35	43	52

[54]	16 AML, 12 NHL, 9 HL, 5 MDS, 4 SAA, 4 CLL, 3 CML, and 2 ALL	Unmanipulated (T cell replete)	Haplo	55	RIC (fludarabine, Cy, TBI, PTCy, tacrolimus, and MMF)	55 PB	96	61	18	28	51	48

[82]	12 AML, 3 CML, 2 ALL, 2 NHL, and 1 HL	Unmanipulated (T cell replete)	Haplo	20	Fludarabine, busulfan ± Cy and PTCy, tacrolimus, and MMF	20 PB	100	30	35	40	50	69

^*∗*^ *p* < 0.05, ^*∗∗*^no statistical comparison available, and ^*∗∗∗*^data not reported.

AML: acute myeloid leukaemia, ALL: acute lymphoblastic leukaemia, HL: Hodgkin's lymphoma, NHL: non-Hodgkin's lymphoma, CLL: chronic lymphocytic leukaemia, CML: chronic myeloid leukaemia, MM: multiple myeloma, SAA: severe aplastic anaemia, MDS: myelodysplastic syndrome, MPD: myeloproliferative disorder, SCD: sickle cell disease, Haplo: haploidentical transplant, MUD: matched unrelated donor transplant, MRD: matched related donor transplant, Sib: matched sibling donor transplant, MMUD: mismatched unrelated donor transplant, RIC: reduced intensity conditioning, MAC: myeloablative conditioning, CNI: calcineurin inhibitor, MMF: mycophenolate mofetil, PTCy: posttransplant cyclophosphamide, Cy: cyclophosphamide, MTX: methotrexate, Me-CCNU: methyl chloride hexamethylene urea nitrate, ATG: antithymocyte globulin, CsA: ciclosporin A, TBI: total body irradiation, OKT3: muromonab CD3, BM: bone marrow, PB: peripheral blood stem cell, CB: cord blood transplant, DLI: donor leucocyte infusion, aGVHD: acute graft-versus-host disease, cGVHD: chronic graft-versus-host disease, TRM: transplant-related mortality, PFS: progression-free survival, DFS: disease-free survival, EFS: event-free survival, and OS: overall survival.
